# Association between blood marker analyses regarding physical fitness levels in Spanish older adults: A cross-sectional study from the PHYSMED project

**DOI:** 10.1371/journal.pone.0206307

**Published:** 2018-10-24

**Authors:** Raquel Aparicio-Ugarriza, Ángel Enrique Díaz, Gonzalo Palacios, María del Mar Bibiloni, Alicia Julibert, Josep Antoni Tur, Marcela González-Gross

**Affiliations:** 1 ImFINE Research Group, Department of Health and Human Performance, Faculty of Physical Activity and Sport Science (INEF), Universidad Politécnica de Madrid, Madrid, Spain; 2 Clinical Laboratory Unit, Department of Sport and Health, Spanish Agency for Health Protection in Sport (AEPSAD), Madrid, Spain; 3 CIBEROBN (Physiopathology of Obesity and Nutrition CB12/03/30038), Madrid, Spain; 4 Research Group on Community Nutrition and Oxidative Stress (NUCOX), University of the Balearic Islands, Palma de Mallorca, Spain; Beijing Key Laboratory of Diabetes Prevention and Research, CHINA

## Abstract

Biomarkers have been postulated as essential variables to measure the effects of exercise on the human body. To investigate the relationship between physical fitness (PF) and blood biomarkers that are associated with disease risk in Spanish older adults, four hundred and twenty-nine adults (57% females) aged older than 55 years from a cross-sectional study were included. A battery of PF test was performed, and participants were divided into 3 groups: low, medium and high fitness. Blood samples were collected, and subjects were also grouped based on a particular biomarker being within its reference range. Furthermore, drug intake and dietary intake were considered for each participant. Higher concentrations out of the reference range were observed for vitamin 25(OH)D (67.9%) and total cholesterol (TC) (58.6%). Participants from the low PF group presented lower significant concentrations out of the reference range for vitamin B_12_ and triglycerides; however, participants in the low PF group showed higher significant concentrations out of the reference range for total homocysteine, creatinine, TC, HDL-cholesterol and LDL-cholesterol (LDL-c) than those in the high PF group (all p<0.05). Considering drugs related to blood lipid modifications, subjects who regularly consumed lipid reducers presented higher significant concentrations out of the reference range for TC and LDL-c than participants who did not take these drugs (p<0.01). Participants from the high PF group presented better blood marker profiles, namely, lower blood markers related to disease risk out of the reference range. These blood markers could be used as a routine method for considering PF groups in older adults.

## Introduction

There is an increasing need to evaluate health-related aspects (i.e., biomarkers) that can be modified by regular physical exercise [1] to provide new evidence and strategies to achieve healthy aging [2]. Aging phenomena are linked with an increased risk of cognitive dysfunction [3] and chronic diseases [4], among other issues. Both factors contribute to changes in motor function and impairments in physical performance in the older adult population. These changes could affect the ability to perform daily activities in this type of population [5].

The pro-healthy effects of regular physical activity (PA) are well-documented [6,7], and previous studies have revealed that physical fitness (PF) is a crucial independent predictor of mortality [8,9]. Attributable risk estimates for all-cause mortality show that low PF, principally low cardiorespiratory fitness and low muscular strength, is a significant risk factor in both sexes [9,10]. PF mixes most of the body functions that are involved in the performance of daily PA [11]. Decreases in PF are related to several negative health effects [12], and biomarkers have been proposed as essential health markers to evaluate the effects of physical exercise on the human body [1,13].

Vitamin deficiencies can cause disorders in behavior, cognitive and emotional status, and personality [14]. Concretely, epidemiologic data indicates that the prevalence of vitamin B_12_ deficiency is from 6% to 40% [15]. Additionally, the worldwide prevalence of pernicious anemia has been estimated to be 24% in older adults (aged over 65 years) [16]. B vitamins and high total homocysteine (tHcy) concentrations have been connected with cardiovascular disease (especially stroke) [17,18], cognitive decline and dementia [14,19,20], fractures [16,21] and mortality [22,23]. Likewise, low vitamin D status may stimulate adipogenesis [24], resulting in further increases in adiposity. Low vitamin D concentrations are also linked with muscle pain, muscle weakness [25,26] and poor PF in older adults [27].

High concentration of low lipoprotein density cholesterol (LDL-c) and low concentration of high lipoprotein density cholesterol (HDL-c) can increase the progression of coronary heart disease [28]. With increasing age, some changes in lipid and lipoprotein concentrations are overall unfavorable [29]. Furthermore, high cardiorespiratory fitness is related to a better blood lipid profile [29].

Through the analysis of different biomarkers, it could be possible to evaluate the reactions of the human body at several levels of PF [1]. However, research regarding the relationship between biomarkers and PF in the older adult population is still ongoing. Hence, the main purpose of this study was to investigate the relationship between blood biomarkers and PF groups associated with disease risk (more blood marker concentrations out of the reference range) in Spanish older adults.

## Methods and materials

### Study design, sample and ethics

The PHYSMED project is a multicenter, cross-sectional study aiming to identify cardiovascular risk factors in sedentary and active older adult subjects. Data were collected from April 2013 to May 2014, and field work took place in 2 Spanish regions: Madrid and Balearic Island. Participants were recruited through health centers, sport federations, sport facilities and municipal clubs located in Madrid and Balearic Island following a snowball system. The study population included 429 adults (57% females) aged 55–88 years. The individual exclusion criteria were as follows: individuals younger than 55 years, those who were institutionalized, and those suffering from a physical or mental illness that would have limited their participation in the physical fitness tests or their ability to respond to the questionnaires or drug intake for clinical research. Each participant signed a written informed consent prior to his/her participation.

The study followed the Declaration of Helsinki 1964 and further amendments. Additionally, the protocol study was approved by the Ethical Committee of the Universidad Politécnica de Madrid.

### Specimen collection and biochemical analyses

Fasted (12 h) blood samples were collected from each participant, and lipid profiles and basic biochemical analyses were performed at the biochemical laboratories of the High Sports Council (Madrid) and Hospital Son Espases & University of Balearic Islands (Palma de Mallorca). The extraction was performed by standard venipuncture using vacuum Vacutainer tubes with separation gel. The tubes were immediately placed on ice, and after coagulum formation, the sample was centrifuged at 3,000 rpm for 10 minutes. Serum total cholesterol (TC), HDL-c, LDL-c, triglycerides (TG), glucose, urea, uric acid, total protein, albumin, glutamic oxalacetic transaminase, glutamic-pyruvic transaminase, gamma glutamyl transpeptidase, iron (FE) and ferritin (FER) were analyzed using a Beckman AU400 analyzer (Beckman AU400, Beckman Instruments, Ltd., Bucks, UK) by photometric methods. Creatinine was analyzed by a colorimetric method (Beckman AU400, Beckman Instruments, Ltd., Bucks, UK). Hematocrit and hemoglobin analyses were performed within the first hour after extraction in EDTA tubes using an automated hematology analyzer (ADVIA 120 Siemens Health Care Diagnostics, SA). These determinations were carried out in each site.

Vitamin B_12_, serum folate (sfolate), red blood cell folate (RBC folate, from EDTA tubes), vitamin D 25[OH]D and tHcy analyses were centralized at the High Sports Council Laboratory in Madrid. Therefore, serum samples from Palma de Mallorca were dry-ice shipped to Madrid and analyzed together with serum samples stored in Madrid at -80 °C, by an electrochemiluminescence method (Elecsys 2010, Roche Diagnostics, IN, USA), except vitamin D 25[OH]D, which was determined using an E411 analyzer (Roche Diagnostics, Switzerland).

### Physical fitness tests

Each participant completed a multicomponent battery of PF tests that have been validated in the older population [30] and in Spanish older adults proposed by Pedrero-Chamizo et al. [31]. Lower body strength was measured by the chair stand test, agility/dynamic balance was performed by the 8-foot up-and-go test, aerobic endurance was assessed by the 6-min walk test, and handgrip strength was measured with a handgrip dynamometer (Takei TKK 5401, Tokyo, Japan, range = 5–100 kg, precision = 0.1 kg) [32]. The handgrip strength was assessed for both hands in a standing position. All tests were performed twice, except the 6-min walk test and the chair stand test, and the best score was retained.

The results of each PF test were stratified by sex and five age groups [divided by five-year periods, except the last group] following the criteria established by Pedrero-Chamizo et al. [31]. The score for each test ranged from 0 (worst) to 3 (best) points. Thus, the maximum score was 12 points. The scores of the PF tests were added together to create a cluster. After that, to classify our population, the PF cluster was divided into 3 different groups: low, medium and high.

### Anthropometric measurements

Weight, body mass index (BMI), total body water (TBW) and fat free mass (FFM) were measured using bioimpedance analysis (TANITA Corp, BC-418MA, Tokyo, Japan) in standardized conditions. Likewise, BMI was calculated as the weight (kg) divided by the square of the height (m). A trained anthropometrist according to the International Society measured waist and hip circumference for the Advancement of Kinanthropometry. Height was assessed to the nearest millimeter using a mobile stadiometer (SECA 213, Germany), with the participant’s head in the Frankfurt plane.

### Dietary assessment

Dietary intake was obtained by means of two 24-h dietary recalls collected on two nonconsecutive days within a period of 2–3 weeks by well-trained dieticians. A computer program was used to convert food into nutrients (ALIMENTA; NUCOX, Palma, Spain) based on Spanish [33,34] and European [35] food composition tables and complemented with food composition data available for Majorcan food items [36].

### Socioeconomic and lifestyle questionnaire

A general questionnaire was used including smoking habits, and participants were grouped in categories as follows: (i) educational level: primary school, secondary school and college-level education; (ii) current income: <600 €/month, 600–900€/month and ≥900 €/month; and (iii) smoker (≥1 cigarette/day) and nonsmoker.

### Medication intake

Participants were asked by means of the EXERNET questionnaire the following question: Do you regularly take drugs? (yes/no). If they answered yes, they were asked about type of medication, manufacturer, frequency of consumption and dose of each drug. Each drug was coded following the Spanish Agency of Medicines and Sanitary Products (https://www.aemps.gob.es/cima/fichasTecnicas.do?metodo=detalleForm). Four groups of drug intake were considered: antidiabetics, renin-angiotensin system drugs, beta blockers and lipid-lowering drugs; all variables were grouped if participants took (coded as 1) or did not take each of the drugs (coded as 0).

### Statistical analysis

Statistics were performed using the statistical software SPSS (IBM Corp. Released 2012. Statistics for Window, V 21.0. Armonk, NY). Descriptive characteristics were summarized by calculating means and standard deviations unless otherwise stated. Each variable was checked for normality of distribution using the Kolmogorov-Smirnov test. The urea, uric acid, total protein, albumin, TC, HDL-c, LDL-c, hemoglobin, hematocrit and vitamin 25[OH]D concentrations showed a normal distribution. The differences between descriptive characteristics and sexes and the differences between biomarkers and PF groups were performed using a one-way ANOVA (for normally distributed variables) and the Kruskal-Wallis test (for nonnormally distributed variables). *Post hoc* analyses were conducted with the Bonferroni adjustment. All variables of the PF tests were checked for normality of distribution by the Kolmogorov-Smirnov test, and these presented a normal distribution.

Regarding vitamin D, the season and the date of blood sample collection were considered for statistical analysis. Samples collected from October to March were classified as “autumn-winter samples”, and those collected from April to September were classified as “spring-summer samples”. The latitudes of each city are similar: Mallorca (39° 34’ N) and Madrid (40° 24’ N).

Likewise, reference ranges established by each laboratory were used to categorize each biomarker as in or out of the reference range (See S1 Table). For vitamin B_12_ and related biomarkers, a different cut-off was applied [15]. Each biomarker within its reference range was coded as 1, and each biomarker out of its reference range was coded as 0.

The association between the status of each biomarker and the PF groups was assessed using the general lineal model. Each biomarker coded as 0 (out of the reference range) was used as a reference in this statistical model. The PF groups were used as an independent variable in which the high PF group was considered the reference. Depending on the biomarkers, drug intake was included into the model as an independent variable in which drug intake was the reference. Moreover, several diet covariables were considered depending on the biomarkers analyzed. The model was not considered when the level of significance of the omnibus test was higher than 0.05. In turn, the lower Akaike information criterion (AIC) was chosen in the final models. Albumin was not analyzed by means of the generalized linear model because only one participant presented abnormal concentrations. Values of p<0.05 were considered statistically significant.

## Results

Table 1 includes the subjects’ characteristics split by PF groups.

**Table 1:** Descriptive characteristics of the studied sample split by PF groups.

	Physical fitness groups			
	Low (n = 131)	Medium (n = 172)	High (n = 126)	p-value
	Mean±SD Median (min-max)	Mean±SD Median (min-max)	Mean±SD Median (min-max)	
**Sex (female)**	76 (58.6)	98 (56.9)	70 (55.6)	>0.05
**Age (y)**	66.8±7.1 66.0 (55.0–87.8)	66.6±6.6 66.0 (55.0–85.4)	66.5±6.2 66.0 (55.1–80.8)	>0.05
**City (Madrid)**	30 (22.6)	83 (47.7)	87 (69.0)	>0.05
**Height (cm)**	161.2±8.8 159.5 (143.0–183.0)	162.2±9.2 160.0 (142.0–184.0)	163.9±8.9 164.0 (145.5–185.5)	<0.05^b^
**Weight (kg)**	72.4±13.5 70.3 (44.9–116.6)	72.9±12.9 71.5 (44.0–99.5)	69.1±11.4 68.2 (46.1–104.6	<0.05^c^
**BMI (kg/m**^**2**^**)**	27.8±3.6 27.4 (17.1–39.1)	27.7±4.1 27.5 (18.4–41.8)	25.6±3.1 25.5 (17.0–33.4)	<0.001 ^a^^,^^b^^,^^c^
**Waist circumference (cm)**	91.6±10.9 91.4 (67.0–121.3)	90.2±11.5 89.9 (61.3–113.7)	86.2±11.2 85.2 (62.7–124.7)	<0.001 ^b^^,^^c^
**Hip circumference (cm)**	102.6±8.1 102.0 (82.7–140.0)	102.5±8.3 102.0 (84–136.8)	99.2±6.7 99.1 (84.0–121.8)	<0.001 ^b^^,^^c^
**Energy (kcal/day)**	1666±486 1618 (540.7–3121)	1650±438 1603 (722–3154)	1778 ±492 1722 (884–3515)	<0.01^b^
**Smoker**	16 (12.0)	6 (3.5)	12 (9.5)	<0.05^a^
**Education**				
Primary school	78 (58.6)	30 (22.6)	25 (18.8)	<0.05 ^a^^,^^b^^,^^c^
Secondary school	68 (39.9)	57 (34.1)	45 (26.0)	
University graduate	40 (31.8)	41 (32.5)	45 (35.7)	
**Current income**				
<600 €/month	43 (32.3)	19 (14.3)	71 (53.4)	<0.001 ^b^^,^^c^
600–900 €/month	41 (24.1)	23 (13.5)	106 (62.4)	
>900 €/month	11 (8.7)	17 (13.5)	98 (77.8)	
**Systolic blood pressure (mm Hg)**	141.0±18.3 138.8 (93.5–219.5)	140.3±17.6 139.3 (100-213-5)	139.6±17.2 139.0 (104.5–185.5)	>0.05
**Diastolic blood pressure (mm Hg)**	81.0±9.8 81.0 (54.5–108.5)	79.4±10.4 79.5 (50.0–126.0)	77.5±8.1 76.5 (58.0–102.0)	<0.05^a^

Data are presented as the means±SD; median (minimum-maximum); n, no. of subjects and values (%). BMI, body mass index. Comparisons between physical fitness groups were analyzed by one-way ANOVA or the Kruskal-Wallis test, according to the normality of the variables. *Post hoc* analyses were conducted with the Bonferroni adjustment.

^a^: Low fitness vs. medium fitness;

^b^: low fitness vs. high fitness;

^c^: medium fitness vs. high fitness.

Level of significance p<0.05.

The descriptive data of biomarkers according to PF groups are shown in Table 2. Blood levels of creatinine (p<0.001), TC (p<0.05), HDL-c (p<0.001), LDL-c (p<0.01), sfolate (p<0.001) and RBC folate (p<0.01) were significantly higher in the high PF group than in the low and medium PF groups. Participants from the high PF group also showed lower significant blood levels than those in the low and medium PF groups for total protein and TG concentrations (both p<0.05).

**Table 2:** Descriptive data of biomarkers divided by PF groups.

Biomarkers	Physical fitness groups						
	Low		Medium		High		
	n	Mean±SD Median (min-max)	n	Mean±SD Median (min-max)	n	Mean±SD Median (min-max)	p-value
**Glucose (mg/dL)**	129	99.5±15.7 96.0 (75.0–190.0)	172	99.1±12.7 98.0 (75.0–144.0)	126	98.1±14.8 97.0 (71.0–181.0)	>0.05
**Urea (mg/dL)**	130	36.3±9.3 35.9 (19.0–69.0)	171	37.5±8.2 37.0 (20.0–59.8)	126	37.5±8.2 37.0 (17.7–62.2)	>0.05
**Uric acid (mg/dL)**	130	5.4±1.3 5.1 (2.8–9.4)	171	5.4±1.4 5.5 (2.6–9.4)	126	5.3±1.3 5.2 (3.0–8.9)	>0.05
**Creatinine (mg/dL)**	131	0.82±0.21 0.78 (0.53–2.18)	172	0.86±0.17 0.84 (0.57–1.46)	126	0.93±0.17 0.91 (0.64–1.41)	<0.001
**Total protein (g/L)**	62	70.4±3.6 70.0 (60.5–81.0)	116	70.3±3.9 70.0 (62.0–81.0)	97	69.0±3.7 69.0 (60.0–78.0)	<0.05
**Albumin (g/L)**	61	42.2±2.1 42.0 (37.7–46.0)	116	42.1±2.1 42.0 (37.0–49.0)	97	42.0±2.5 42.0 (35.0–48.0)	>0.05
**TC (mg/dL)**	130	204.0±35.3 198.5 (112.0–293.0)	172	210.8±34.6 206.0 (141.0–351.0)	126	218.4±33.3 219.0 (131.0–321.0)	<0.01
**HDL-c (mg/dL)**	129	53.0±12.6 53.0 (20.0–91.0)	169	55.8±13.1 54.5 (27.7–95.2)	124	59.2±13.5 57.9 (32.6–98.0)	<0.001
**LDL-c (mg/dL)**	129	129.7±30.1 126.0 (69.4–218.8)	169	135.1±29.6 134.0 (69.0–249.5)	124	141.3±27.4 142.7 (71.4–224.8)	<0.01
**TG (mg/dL)**	130	104.3±38.4 97.2 (33.0–208.0)	172	99.1±40.8 90.1 (45.2–268.0)	126	93.9±37.9 84.1 (40.6–274.1)	<0.05
**Hematocrit (%)**	131	43.4	168	43.3	125	43.1	>0.05
**Hemoglobin (g/dL)**	131	14.6±1.2 14.7 (12.0–17.0)	168	14.5±1.1 14.3 (12.0–18.0)	125	14.5±1.0 14.6 (12.0–17.0)	>0.05
**FE (ug/dL)**	61	93.1±23.8 94.0 (38.2–137.5)	115	91.6±33.9 87.5 (38.3–331.0)	98	93.8±28.2 90.7 (45.4–201.2)	>0.05
**FER (ng/dL)**	60	122.3±101.0 110.7 (12.0–671.5)	115	125.5±99.5 93.6 (13.0–542.0)	96	126.6±93.4 98.0 (16.8–419.3)	>0.05
**tHcy (μmol /dL)**	111	13.4±4.6 12.8 (1.8–35.5)	164	12.9±4.4 12.2 (2.0–31.3)	124	12.3±4.0 11.6 (3.9–34.2)	= 0.090
**Vitamin B**_**12**_ **(pg/ mL)**	109	375.8±181.2 345.3 (86.5–1163.0)	163	390.3±202.0 343.7 (122.8–1650.0)	124	386.4±172.4 350.2 (100.0–1402.0)	>0.05
**sfolate (ng/ mL)**	111	10.1±4.2 8.8 (2.7–32.7)	164	11.1±4.4 10.4 (3.9–29.3)	124	11.9±4.2 11.7 (4.7–29.9)	<0.001
**RBC folate (ng/ mL)**	91	345.9±117.2 326.9 (156.1–728.8)	133	369.2±114.5 351.2 (184.2–862.6)	113	384.2±105.5 379.1 (149.0–710.0)	<0.01
**Vitamin D (25(OH)D) (mg/mL)**	109	25.6±10.3 24.1 (4.5–51.9)	154	25.9±9.8 25.4 (4.3–64.4)	120	26.0±10.5 25.5 (6.7–57.7)	>0.05

Values are presented as the means±SD; n, median (minimum-maximum); no. of subjects. sfolate, serum folate; RBC folate; red blood cell folate; tHcy, total homocysteine; TC, total cholesterol; TG, triglycerides; HDL-c, HDL-cholesterol; LDL-c, LDL-cholesterol; FE, iron, FER, ferritin. Comparisons between physical fitness groups were analyzed by one-way ANOVA or the Kruskal-Wallis test, according to the normality of the variables. Level of significance p<0.05.

**Fig 1:**
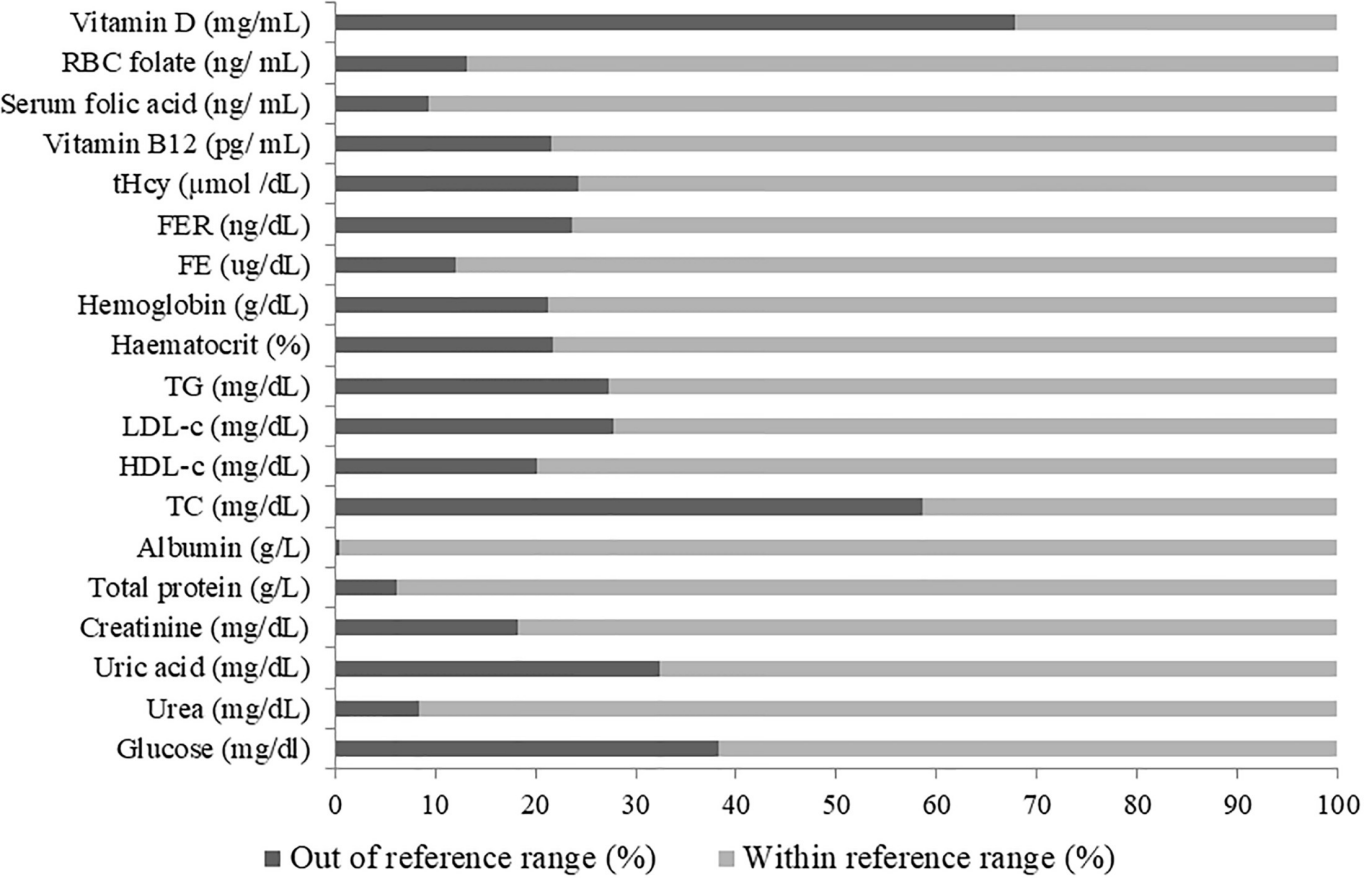
Percentage of each biomarker in and out of the reference range. Values are presented as percentages (%). sfolate, serum folate; tHcy, total homocysteine; TC, total cholesterol; TG, triglycerides; HDL-c, HDL-cholesterol; LDL-c, LDL-cholesterol; FE, iron, FER, ferritin.

Fig 1 displays the percentage of each biomarker in and out of the reference range. A total of 67.9% and 58.6% of the sample presented low vitamin 25[OH]D concentrations and high TC concentrations, respectively, that were out of the reference range. Likewise, several blood markers including glucose (38.3%), uric acid (32.3%), LDL-c (27.7%) and tHcy (4.3%) that were higher than the reference range.

Results from the general linear model investigating the associations between blood biomarkers and PF groups are shown in Table 3. Participants from the low PF group had lower vitamin B_12_ concentrations out of the reference range (both models, p<0.05). However, participants in the low PF group showed greater tHcy concentrations out of the reference range than those from the high PF group (both models, p<0.05). Furthermore, vitamin B_12_ deficiency and tHcy concentrations in males increased significantly in parallel with advanced age (p<0.05). Likewise, high total protein concentrations out of reference range were observed in the low PF (both models, p>0.05) and medium PF groups (both model, p>0.05) compared to the high PF group. Participants from the low PF group also had a higher likelihood of having creatinine concentrations out of the reference range than those from the high PF group (both models, p<0.001).

**Table 3:** Association between biomarkers and physical fitness.

**Dependent variable: Creatinine (mg/dL)**					
**Model 1**	**β**	**p-value**	**Model 2**	**β**	**p-value**
Male	1.431	0.000	Male	1.668	0.025
Female	0.000		Female	0.000	
Age (y)	0.017	0.402	Age (y)	0.017	0.433
Low fitness	-1.323	0.000	Weight (kg)	0.018	0.450
Medium fitness	-0.696	0.062	Height (cm)	0.009	0.763
High fitness	0.000		FFM (kg)	-0.035	0.560
Protein intake (g)	0.007	0.288	Low fitness	-1.381	0.000
			Medium fitness	-0.727	0.057
			High fitness	0.000	
			Protein intake (g)	0.008	0.277
**Dependent variable: Total protein (g/L)**					
**Model 1**	**β**	**p-value**	**Model 2**	**β**	**p-value**
Male	0.805	0.195	Male	0.607	0.360
Female	0.000		Female	0.000	
Age (y)	0.005	0.894	Age (y)	0.038	0.364
Low fitness	2.002	0.061	Low fitness	2.058	0.063
Medium fitness	1.039	0.069	Medium fitness	1.048	0.082
High fitness	0.000		High fitness	0.000	
Protein intake (g)	0.026	0.737	GOT (U/L)	-0.013	0.846
Vegetal intake (g)	-0.050	0.539	GPT (U/L)	0.030	0.066
Animal intake (g)	0.004	0.955	GGT (U/L)	-0.001	0.963
**Dependent variable: Total cholesterol (mg/dL)**					
**Model 1**	**β**	**p-value**	**Model 2**	**β**	**p-value**
Male	1.092	0.000	Male	1.110	0.000
Female	0.000		Female	0.000	
Age (y)	0.023	0.151	Age (y)	0.025	0.125
Low fitness	1.028	0.000	Low fitness	1.036	0.000
Medium fitness	0.546	0.038	Medium fitness	0.600	0.025
High fitness	0.000		High fitness	0.000	
Total cholesterol intake (mg)	0.000	0.473	Total cholesterol intake (mg)	0.000	0.629
			SFA intake (g)	-0.106	0.037
No lipid-lowering drugs	-0.962	0.000	MUFA intake (g)	-0.109	0.033
Yes lipid-lowering drugs	0.000		PUFA intake (g)	-0.148	0.025
			Lipid intake (g)	0.103	0.021
			No lipid-lowering drugs	-0.973	0.000
			Yes lipid-lowering drugs	0.000	
**Dependent variable: HDL-cholesterol (mg/dL)**					
**Model 1**	**β**	**p-value**	**Model 2**	**β**	**p-value**
Male	-1.829	0.000	Male	-1.964	0.000
Female	0.000		Female	0.000	
Age (y)	0.002	0.910	Age (y)	0.007	0.701
Low fitness	-0.901	0.007	Low fitness	-0.917	0.007
Medium fitness	-0.391	0.231	Medium fitness	-0.384	0.246
High fitness	0.000		High fitness	0.000	
Total cholesterol intake (mg)	-0.001	0.375	Total cholesterol intake (mg)	-0.001	0.172
No lipid-lowering drugs	-0.148	0.625	SFA intake (g)	0.091	0.119
Yes lipid-lowering drugs	0.000		MUFA intake (g)	0.068	0.225
			PUFA intake (g)	0.025	0.723
			Lipid intake (g)	-0.054	0.272
			No lipid-lowering drugs	-0.216	0.486
			Yes lipid-lowering drugs	0.000	
**Dependent variable: LDL-cholesterol (mg/dL)**					
**Model 1**	**β**	**p-value**	**Model 2**	**β**	**p-value**
Male	0.297	0.158	Male	0.240	0.262
Female	0.000		Female	0.000	
Age (y)	0.026	0.132	Age (y)	0.032	0.078
Low fitness	0.563	0.051	Low fitness	0.651	0.029
Medium fitness	0.488	0.065	Medium fitness	0.589	0.030
High fitness	0.000		High fitness	0.000	
Total cholesterol intake (mg)	0.237	0.180	Total cholesterol intake (mg)	-0.002	0.064
No lipid-lowering drugs	-1.090	0.001	SFA intake (g)	-0.079	0.165
Yes lipid-lowering drugs	0.000		MUFA intake (g)	-0.129	0.024
			PUFA intake (g)	-0.015	0.904
			Lipid intake (g)	0.009	0.061
			No lipid-lowering drugs	-1.130	0.001
			Yes lipid-lowering drugs	0.000	
**Dependent variable: Triglycerides (mg/dL)**					
**Model 1**	**β**	**p-value**	**Model 2**	**β**	**p-value**
Male	-0.436	0.056	Male	-0.557	0.022
Female	0.000		Female	0.000	
Age (y)	0.026	0.132	Age (y)	0.006	0.745
Low fitness	-0.720	0.013	Low fitness	-0.694	0.020
Medium fitness	-0.387	0.170	Medium fitness	-0.312	0.274
High fitness	0.000		High fitness	0.000	
Total cholesterol intake (mg)	0.000	0.803	Total cholesterol intake (mg)	-0.001	0.209
No lipid-lowering drugs	0.437	0.079	SFA intake (g)	-0.116	0.053
Yes lipid-lowering drugs	0.000		MUFA intake (g)	-0.166	0.007
			PUFA intake (g)	-0.123	0.104
			Lipid intake (g)	0.141	0.009
			No lipid-lowering drugs	0.423	0.095
			Yes lipid-lowering drugs	0.000	
**Dependent variable: Total homocysteine (μmol /dL)**					
**Model 1**	**β**	**p-value**	**Model 2**	**β**	**p-value**
Male	-1.284	0.000	Male	-1.287	0.000
Female	0.000		Female	0.000	
Age (y)	-0.046	0.014	Age (y)	-0.047	0.014
Low fitness	-0.723	0.030	Low fitness	-0.716	0.033
Medium fitness	-0.334	0.283	Medium fitness	-0.329	0.285
High fitness	0.000		High fitness	0.000	
Vitamin B_12_ intake (ug)	0.035	0.080	Vitamin B_12_ intake (ug)	0.034	0.113
			Folate intake (ug)		
Yes Renin-angiotensin system drug	0.542	0.053	Yes Renin-angiotensin system drug	0.541	0.053
No Renin-angiotensin system drug	0.000		No Renin-angiotensin system drug	0.000	
Yes beta blocker	0.352	0.445	Yes beta blocker	0.355	0.442
No beta blocker	0.000		No beta blocker	0.000	
**Dependent variable: Vitamin B**_**12**_ **(pg/mL)**					
**Model 1**	**β**	**p-value**	**Model 2**	**β**	**p-value**
Male	-0.747	0.004	Male	-0.749	0.004
Female	0.000		Female	0.000	
Age (y)	-0.053	0.006	Age (y)	-0.053	0.005
Low fitness	-0.847	0.020	Low fitness	-0.757	0.028
Medium fitness	-0.509	0.099	Medium fitness	-0.539	0.091
High fitness	0.000		High fitness	0.000	
Protein intake (g)	0.005	0.468	Vitamin B_12_ intake (ug)	0.019	0.265
			Folate intake (ug)	0.000	0.692
**Dependent variable: Glucose (mg/dL)**					
**Model 1**	**β**	**p-value**	**Model 2**	**β**	**p-value**
Male	-10.962	0.002	Male	-1.738	0.015
Female	0.000		Female	0.000	
Age (y)	-0.019	0.245	Age (y)	-0.012	0.492
Low fitness	-0.433	0.282	Low fitness	-0.275	0.513
Medium fitness	-0.307	0423	Medium fitness	-0.022	0.957
High fitness	0.000		High fitness	0.000	
Carbohydrate intake (g)	0.000	0.896	Weight (kg)	0.064	0.185
Mono & Disaccharides intake (g)	0.004	0.616	BMI (kg/m^2^)	-0.227	0.011
Polysaccharides intake (g)	-0.002	0.756	FFM (kg)	-6-323	0.108
			TBW (KG)	8.634	0.108
Yes antidiabetic drug	4.366	0.000	Yes antidiabetic drug	4.339	0.000
No antidiabetic drug	0.000		No antidiabetic drug	0.000	
**Dependent variable: Uric acid (mg/dL)**					
**Model 1**	**β**	**p-value**			
Male	-2.249	0.000			
Female	0.000				
Age (y)	-0.005	0.783			
Low fitness	-0.135	0.663			
Medium fitness	-0.420	0.145			
High fitness	0.000				
Protein intake (g)	0.006	0.261			
**Dependent variable: Urea (mg/dL)**					
**Model 1**	**β**	**p-value**			
Male	-0.650	0.096			
Female	0.000				
Age (y)	-0.038	0.165			
Low fitness	-0.213	0.639			
Medium fitness	0.059	0.897			
High fitness	0.000				
Protein intake (g)	-0.018	0.005			
**Dependent variable: Hematocrit (%)**					
**Model 1**	**β**	**p-value**	**Model 2**	**β**	**p-value**
Male	-2.568	0.000	Male	-1.746	0.000
Female	0.000		Female	0.000	
Low fitness	-0.429	0.249	Low fitness	-0.608	0.195
Medium fitness	-0.480	0.134	Medium fitness	-0.497	0.211
High fitness	0.000		High fitness	0.000	
			Red blood cells (10^6/mm3)	-5.559	0.000
**Dependent variable: Hemoglobin (g/dL)**					
**Model 1**	**β**	**p-value**	**Model 2**	**β**	**p-value**
Male	-2.408	0.000	Male	-1.677	0.000
Female	0.000		Female	0.000	
Age (y)	0.029	0.157	Age (y)	0.028	0.221
Low fitness	-0.575	0.100	Low fitness	-0.527	0.171
Medium fitness	-0.433	0.192	Medium fitness	-0.434	0.232
High fitness	0.000		High fitness	0.000	
			Red blood cells (10^6/mm3)	-3.137	0.000
**Dependent variable: Iron (ug/dL)**					
**Model 1**	**β**	**p-value**	**Model 2**	**β**	**p-value**
Male	0.405	0.307	Male	0.432	0.293
Female	0.000		Female	0.000	
Age (y)	-0.010	0.714	Age (y)	-0.012	0.660
Low fitness	-0.176	0.729	Low fitness	-0.326	0.528
Medium fitness	-0.069	0.874	Medium fitness	-0.204	0.646
High fitness	0.000		High fitness	0.000	
Iron intake (mg)	-0.008	0.163	B_12_ intake (mg)	0.010	0.714
			Folate intake (ug)	-0.003	0.033
			Iron intake (mg)	-0.008	0.152
**Dependent variable: Ferritin (ng/dL)**					
**Model 1**	**β**	**p-value**	**Model 2**	**β**	**p-value**
Male	-1.310	0.000	Male	-1.336	0.000
Female	0.000		Female	0.000	
Age (y)	-0.001	0.951	Age (y)	0.000	0.986
Low fitness	0.184	0.665	Low fitness	0.211	0.626
Medium fitness	0.067	0.843	Medium fitness	0.073	0.831
High fitness	0.000		High fitness	0.000	
Iron intake (mg)	0.032	0.114	B_12_ intake (mg)	0.010	0.641
			Folate intake (ug)	0.000	0.858
			Iron intake (mg)	0.029	0.149
			Lipid intake (g)	-0.053	0.284
**Dependent variable: Serum folate (ng/mL)**					
**Model 1**	**β**	**p-value**	**Model 2**	**β**	**p-value**
Male	0.202	0.581	Male	0.130	0.725
Female	0.000		Female	0.000	
Age (y)	0.013	0.651	Age (y)	0.015	0.586
Low fitness	0.658	0.173	Low fitness	0.632	0.192
Medium fitness	0.343	0.387	Medium fitness	0.372	0.350
High fitness	0.000		High fitness	0.000	
Folate intake (ug)	-0.001	0.240	Folate intake (ug)	-0.001	0.202
			Vitamin B_12_ intake (ug)	0.030	0.368
**Dependent variable: RBC folate (ng/mL)**					
**Model 1**	**β**	**p-value**	**Model 2**	**β**	**p-value**
Male	0.784	0.034	Male	0.730	0.051
Female	0.000		Female	0.000	
Age (y)	0.018	0.479	Age (y)	0.019	0.440
Low fitness	0.051	0.904	Low fitness	0.017	0.967
Medium fitness	0.086	0.826	Medium fitness	0.097	0.803
High fitness	0.000		High fitness	0.000	
Folate intake (ug)	-0.001	0.404	Folate intake (ug)	-0.001	0.358
			Vitamin B_12_ intake (ug)	0.017	0.496
**Dependent variable: Vitamin D 25[OH]D (ng/mL)**					
**Model 1**	**β**	**p-value**			
Male	0.235	0.300			
Female	0.000				
Age (y)	-0.016	0.351			
Low fitness	0.221	0.442			
Medium fitness	0.101	0.705			
High fitness	0.000				
Vitamin D intake (ug)	0.005	0.318			
Calcium intake (mg)	0.000	0.688			

β; standardized coefficients. BMI; body mass index; FFM, fat free mass; SFA; saturated fatty acids; MUFA; mono-unsaturated fatty acids; PUFA; polyunsaturated fatty acids; GOT; Glutamic oxalacetic transaminase, GPT; Glutamic-pyruvic transaminase, GGT; Gamma glutamyl transpeptidase; RBC folate, red blood cell folate; TBW, total body water. Level of significance p<0.05.

Concerning the blood lipid profile, more participants in the low PF group than in the high PF group had TC levels above the reference range (both models, p<0.001). Males had higher concentrations of TC than females (p<0.001). Additionally, when considering lipid and fatty acid intake in the model, these associations remained significant (p<0.05). Similarly, participants from the low (both models, p<0.001) and medium (both models, p<0.05) PF groups had greater HDL-c concentrations out of the reference range than those from the high PF group. Considering drugs related to blood lipid modifications, participants who regularly consumed lipid-reducing drugs presented higher concentrations out of the reference range for TC and LDL-c than participants who did not take these drugs (both models, p<0.01). In contrast, more individuals in the high PF group than in the low PF group had TG concentrations out of the reference range (both models, p<0.05), and this significant association remained when lipids and monounsaturated fatty acids were considered as cofounders (model 2, p<0.05).

## Discussion

This study has shown that there are significant associations between PF levels and several blood biomarkers related to disease risk. Our main results revealed that participants from the low PF group showed lower vitamin B_12_ and TG concentrations out of the reference range and higher concentrations out of the reference range for tHcy, creatinine, TC, HDL-c and LDL-c. A high percentage of the sample population presented also with low vitamin 25[OH]D and high TC, glucose, uric acid, LDL-c and tHcy concentrations independent of PF. To the best of the authors´ knowledge, there are no studies analyzing the relationship between blood biomarker concentrations within and out of the reference range and PF groups in Spanish older adults through this holistic approach. In this sense, participants who showed concentrations out of the reference range were also considered as having concentrations related to disease risk because long periods with biomarker levels out of the reference range could have a negative effect on health.

Regarding B-vitamins, subclinical deficiency and deficiency of these vitamins are common in the older adult population [15,37], and both tend to increase with age [38]. A total of 51.8% of the studied population presented subclinical deficiency considering as a cut-off point a concentration less than 352.3 pg/mL [15] (data not shown). Severe vitamin B_12_ deficiency causes an irreversible degeneration of the nervous system. Vitamin B_12_ and folate are involved as coenzymes of numerous regulating enzymes [39].

The relationships among vitamin B_12_, sfolate, RBC folate and tHcy are an ongoing research areas, especially if PF and PA are considered [1]. Likewise, no single blood marker has been described that could effectively evaluate the aging process [40]. Surprisingly, participants from the low PF group had lower vitamin B_12_ concentrations out of the reference range. Additionally, participants included in the low PF group presented significantly high tHcy concentrations out of the reference range, but no association was found among sfolate and RBC folate and PF levels in our study. Joubert et al. indicated that participants from the active and high PF groups presented a significantly higher concentration of tHcy than the sedentary group, as measured by means of treadmill VO_2 max_ [41]. Alomari et al. observed an inverse association between PA levels and tHcy. Additionally, they reported a lower concentration of tHcy in subjects with higher vs. lower PA adjusting for vitamin B_12_ [42]. In this sense, Murakami et al. also did not observe differences in tHcy after controlling for age, sex, and folate intake according to PA groups [43]. In our study, with advancing age (in males), vitamin B_12_ deficiency and tHcy concentrations increased. Kuo et al. observed that high tHcy concentrations were inversely associated with cardiovascular fitness in females but not in males [44]. Likewise, Schoor et al. observed that females in the highest quartile of tHcy had a significantly lower PF than those in the lowest quartile [12]. A recent systematic review suggested positive results of increasing folic acid and vitamin B_12_ supplementation and regular physical exercise to prevent hyperhomocysteinemia [45].

Vitamin D deficiency has been previously published in all age stages [24,46,47] and is still an unsolved public health problem. In our population, a total of 67.9% of participants presented vitamin D concentrations below 29.99 ng/mL. Vitamin D is considered a marker of improved muscle condition, mainly in healthy older adults with lower PA as measured by means of PA questionnaires [27]. Al-Eisa et al. found that participants with low PA showed significantly lower 25[OH]D serum concentrations than active participants [27]. However, in our study there was no association between PF groups and vitamin D.

Another interesting finding in our study was that the low PF group presented a higher risk of high TC, high LDL-c, low HDL-c and low TG concentrations out of the reference ranges than the high PF group. These results are in accordance with the findings of other authors [48,49]. Furthermore, LDL-c and TG showed a significant relationship with PF groups when lipid intake was included into the statistical analysis but not with TC intake. These results confirm the relationship between lipid intake suggested by Siri-Tarino et al. [50]. Dvorak et al. observed that older adult subjects with high cardiorespiratory fitness, independent of their PA levels, presented lower concentrations of TG, TC, total HDL-c and low LDL-c [51].

In our study, uric acid and urea were analyzed, but there were not significant differences between PF groups, and there was also no association between PF and concentrations out of the reference range. These results could suggest that both blood biomarkers might increase after intensive exercise [1,52,53] but not as a consequence of performed PA during their lives. Low concentrations of total protein are associated with malnutrition [54]. A significant relationship was obtained between total protein and PF groups, and a major tendency for total protein concentrations out of the reference range was observed between low and medium PF groups compared to the high PF group. Furthermore, participants in the low PF group showed a significantly higher risk of presenting concentrations out of the reference range for creatinine than participants in the high PF group in our study.

Hematocrit, hemoglobin and red blood cells have been related to significant higher possibilities of adverse health-status measurements (i.e., multiple morbidities, cognitive impairment, disability and mortality) [55]. Nevertheless, in our study, we could not find these associations between hematocrit and hemoglobin and PF groups. This could be because similar concentrations were obtained in all PF groups. Low hematocrits have been observed in trained persons mainly due to an increased plasma volume [56].

Several biomarkers are key in biological pathways and have been related to cardiovascular disease, and it is clearly established that recurrent exercise modifies a wide number of blood biomarkers. Dietary intake was considered in the analysis due to its fundamental role in decreasing the progression of chronic disease [57].

The current study has some limitations. First, the cross-sectional design cannot determine the cause-effect relationship between PF and biomarkers. Second, all participants were volunteers; thus, the outcomes are applicable only for the study group. Despite the abovementioned limitations, the PHYSMED project has several strengths. Likewise, some results are well known (e.g., lipid markers); this study analyzed several blood markers, including PF as a health marker. Another strength is the inclusion of a battery of PF tests, which is a more objective and precise method than questionnaires. Additionally, clustering of PF produces an alternative approach to summarizing PF levels, and it allows a realistic, wide vision about behavioral patterns. Moreover, the strict standardization of the field work and the blood sample protocol among the cities that took part in the study was another strength.

In conclusion, the study shows new approaches regarding the relationship between blood biomarkers and different groups of PF. Blood markers of health were generally associated with high PF in Spanish older adults. Regular physical exercise modifies a broad variety of metabolic processes that are then reflected by particular variations in biomarkers; therefore, both PF and blood markers should be considered when analyzing health status in older subjects. The proposed blood markers could be used routinely considering also the PF groups, and public health strategies should be implemented regarding biomarkers at risk. Additionally, longitudinal studies are needed to predict the capabilities of blood markers according to PF.

## Supporting information

S1 Table Description of the reference ranges used in each biomarker. HDL-Cholesterol; high density lipoprotein cholesterol. LDL-cholesterol; low density lipoprotein cholesterol.
(DOCX)
