# Supplementary material for: Association between blood marker analyses regarding physical fitness levels in Spanish older adults: A cross-sectional study from the PHYSMED project
Source: PLoS One. 2018 Oct 24;13(10):e0206307. doi: 10.1371/journal.pone.0206307 (PMC6200257; doi:10.1371/journal.pone.0206307)
Supplement: S1 Table — HDL-Cholesterol; high density lipoprotein cholesterol. LDL-cholesterol; low density lipoprotein cholesterol. (DOCX) [file pone.0206307.s001.docx]

**S1 Table.** Description of the reference ranges used in each biomarker.

| **Components** | **Description** | **Score** |
| --- | --- | --- |
| Glucose | Reference value: ≥ 110.0 mg/dL | ≥ 110.0 mg/dL= 0 points  < 110.0 mg/dL= 1 point |
| Urea | Reference value: ≥ 50.0 mg/dL | ≥ 50.0 mg/dL= 0 points  < 50.0 mg/dL= 1 point |
| Uric acid | Reference value: ≥ 5.9 mg/dL | ≥ 5.9 mg/dL= 0 points  < 5.9 mg/dL= 1 point |
| Creatinine | Reference value: ≥ 1.4 mg/dL males; ≥ 1.3 mg/dL females | ≥ 1.4 mg/dL males; ≥ 1.3 mg/dL females= 0 points  < 1.4 mg/dL males; < 1.3 mg/dL females= 1 point |
| Total protein | Reference value: ≤ 6.4 g/dL | ≤ 6.4 g/dL= 0 points  > 6.4 g/dL = 1 point |
| Albumin | Reference value: ≤ 3.0 g/dL | ≤ 3.0 g/dL= 0 points  > 3.0 g/dL= 1 point |
| Total cholesterol | Reference value: ≥ 200.0 mg/dL | ≥ 200.0 mg/dL= 0 points  < 200.0 mg/dL= 1 point |
| HDL-cholesterol | Reference value: ≤ 40.0 mg/dL males;  ≤ 50.0 mg/dL females | ≤ 40.0 mg/dL males; ≤ 50.0 mg/dL females= 0 points  > 40.0 mg/dL males; > 50.0 mg/dL females= 1 point |
| LDL-cholesterol | Reference value: ≥ 150.0 mg/dL | ≥ 150.0 mg/dL= 0 points  < 150.0 mg/dL= 1 point |
| Triglycerides | Reference value: ≥ 150.0 mg/dL | ≥ 150.0 mg/dL= 0 points  < 150.0 mg/dL= 1 point |
| Haematocrit | Reference value: ≤ 35% | ≤ 35%= 0 points  > 35%= 1 point |
| Hemoglobin | Reference value: ≤ 15.5 g/dL | ≤ 15.5 g/dL= 0 points  > 15.5 g/dL= 1 point |
| Iron | Reference value: ≤ 60.0 µg/dL | ≤ 60.0 µg/dL= 0 points  > 60.0 µg/dL = 1 point |
| Ferritin | Reference value: ≤ 30.0 µg/dL | ≤ 30.0 µg/dL= 0 points  > 30.0 µg/dL= 1 point |
| Total homocysteine | Reference value: ≥ 15.0 µmol/L | ≥ 15.0 µmol/L= 0 points  < 15.0 µmol/L= 1 point |
| Vitamin B_12_ | Reference value: ≤ 250.0 pg/mL | ≤ 250.0 pg/mL= 0 points  > 250.0 pg/mL= 1 point |
| Serum folate | Reference value: ≤ 6.8 nmol/L | ≤ 6.8 nmol/L= 0 points  > 6.8 nmol/L= 1 point |
| Red blood cell folate | Reference value: ≤ 212.0 ng/mL | ≤ 212.0 ng/mL= 0 points  > 212.0 ng/mL= 1 point |
| Vitamin D | Reference value: ≤ 20.0 ng/mL | ≤ 20.0 ng/mL= 0 points  > 20.0 ng/mL= 1 point |

HDL-Cholesterol; high density lipoprotein cholesterol. LDL-cholesterol; low density lipoprotein cholesterol.
